# Supplementary material for: Unaware processing of words activates experience-derived information in conceptual-semantic brain networks
Source: Imaging Neurosci (Camb). 2025 Feb 26;3:imag_a_00484. doi: 10.1162/imag_a_00484 (PMC12319976; doi:10.1162/imag_a_00484)
Supplement: Supplementary Material [file imag_a_00484-supp.pdf]

Supplementary materials for:

Unaware Processing of Words Activates Experience-Derived Information in Conceptual-Semantic Brain Networks

Marta Ghio[1,2,*], Barbara Cassone[3,*], Marco Tettamanti[2,3]

[1] Faculty of Mathematics and Natural Sciences, Heinrich Heine University Düsseldorf, Düsseldorf, Germany.

[2] CIMeC - Center for Mind/Brain Sciences, University of Trento, Italy.

[3] Department of Psychology, University of Milan-Bicocca, Milano, Italy.

[*] Equal contribution

Corresponding author:

Marco Tettamanti

Department of Psychology, University of Milan-Bicocca, Milano, Italy

Piazza dell'Ateneo Nuovo 1, I-20126 Milano, Italy

Email: marcodante.tettamanti@unimib.it

Supplementary Table 1A. Lexical and sublexical variables of experimental stimuli.

Word stimuli	Word Type	N. orth. neigh.	Lex. freq.	First bigram freq.	Medial bigrams freq.	Last bigram freq.	Mean bigram freq.	N. syllables	N. letters	Visual length (mm)
accendino	MO	2	557	12476	81489.50	133168	75711.17	4	9	32.68
anello	MO	5	5515	42551	82939	34588	53359.33	3	6	21.51
annaffiatoio	MO	NaN	12	42551	52265	52265	49027	5	12	40.72
apriscatole	MO	0	62	10873	80625.25	73827	55108.42	5	11	38.44
spada	MO	4	4367	19719	20830.50	10909	17152.83	2	5	18.21
bastone	MO	5	1654	14724	101660.25	70433	62272.42	3	7	26.16
bicchiere	MO	1	3888	6547	80262.67	133974	73594.56	3	9	29.72
bisturi	MO	0	447	6547	64315	35748	35536.67	3	7	21.93
bollitore	MO	1	121	6010	97511.83	133974	79165.28	4	9	30.73
bottiglia	MO	1	4283	6010	63278.67	57191	42159.89	3	9	28.87
bottone	MO	3	952	6010	102066.50	70433	59503.17	3	7	26.16
braccialetto	MO	1	751	5011	76116.78	223642	101589.9267	4	12	41.99
bullone	MO	2	78	4458	92656.50	70433	55849.17	3	7	25.32
cacciavite	MO	1	312	46052	54964	86456	62490.67	4	10	33.1
caffettiera	MO	1	107	46052	70069.75	65653	60591.58	4	11	37.59
calcolatrice	MO	0	118	46052	88551.67	18061	50888.22	5	12	41.83
campanello	MO	2	877	46052	77158.71	34588	52599.57	4	10	37.17
cavatappi	MO	1	102	46052	75488.67	3639	41726.56	4	9	30.82
cellulare	MO	1	7074	14848	92517	133168	80177.67	4	9	31.58
cerniera	MO	1	213	14848	103106.60	65653	61202.53	3	8	28
chitarra	MO	1	1421	81739	87567.40	65653	78319.80	3	8	27.86
coltello	MO	3	4712	135447	86921.80	34588	85652.27	3	8	28.36
compasso	MO	2	35	135447	53220.20	40259	76308.73	3	8	31.5
computer	MO	0	8130	135447	63069	48415	82310.33	3	8	31.07
coperchio	MO	0	293	135447	61852.17	52265	83188.06	3	9	32.94
cucchiaio	MO	0	575	10568	39132.50	52265	33988.50	3	9	30.65
fazzoletto	MO	1	708	33631	55289.29	223642	104187.43	4	10	35.9
fiammifero	MO	1	330	25684	59668.43	48641	44664.48	4	10	34.46
forbici	MO	1	677	17526	69147.25	14195	33622.75	3	7	22.44
forchetta	MO	1	394	17526	70944.17	93005	60491.72	3	9	33.36
fucile	MO	5	3590	9500	40289	73827	41205.33	3	6	19.3
grattugia	MO	0	45	14632	88826.67	57191	53549.89	3	9	32
grilletto	MO	0	1584	14632	85315.83	223642	107863.28	3	9	30.65
interruttore	MO	1	652	107539	93962.89	133974	111825.30	5	12	43.01
manette	MO	2	1368	56674	97576	86456	80235.33	3	7	25.91
manganello	MO	1	99	56674	89747.43	34588	60336.48	4	10	37.76
maniglia	MO	2	306	56674	71121.60	57191	61662.20	3	8	26.33
mannaia	MO	1	70	56674	66843.75	57191	60236.25	3	7	24.8
manopola	MO	2	111	56674	69067	78467	68069.33	4	8	30.9
martello	MO	5	1141	56674	98616.60	34588	63292.87	3	8	29.38
matita	MO	4	559	56674	129059.33	93005	92912.78	3	6	20.24
mestolo	MO	2	63	28080	90120	34588	50929.33	3	7	26.59
orologio	MO	0	4034	13108	48697.60	52265	38023.53333	4	8	29.72

padella	MO	6	368	45016	69713.50	78467	64398.83	3	7	22.5
pennarello	MO	1	159	74312	105621.86	34588	71507.29	4	10	36.49
pennello	MO	3	335	74312	95097.40	34588	67999.13	3	8	29
pentola	MO	5	492	74312	126565.25	78467	93114.75	3	7	25.5
pettine	MO	4	233	74312	94042	70433	79595.67	3	7	23.28
pinzatrice	MO	NaN	47	33910	91263.14	18061	47744.71	4	10	32.6
portafoglio	MO	0	1513	42994	58623.13	52265	51294.04	4	11	39.54
pugnale	MO	1	859	13830	45160	73827	44272.33	3	7	25.82
pulsante	MO	1	1066	13830	88011	86456	62765.67	3	8	28.79
racchetta	MO	3	148	16575	57153.33	93005	55577.78	3	9	33.3
rasoio	MO	0	569	16575	23392.67	52265	30744.22	3	6	21.25
rastrello	MO	1	72	16575	92033.83	34588	47732.28	3	9	32.26
righello	MO	0	69	51082	53767.80	34588	46479.27	3	8	27.6
schiaccianoci	MO	0	72	25029	62087.80	14195	33770.60	4	13	43.6
scolapasta	MO	0	8	25029	56137.86	93005	58057.29	4	10	36.49
siringa	MO	1	417	50691	64356.75	5231	40092.92	3	7	22.8
spatola	MO	1	63	19719	92287	78467	63491	3	7	25.3
spazzola	MO	2	248	19719	36734.20	78467	44973.40	3	8	28.62
spazzolino	MO	1	479	19719	51548.43	133168	68145.14	4	10	34.71
spremiagrumi	MO	NaN	15	19719	37193.33	9105	22005.78	5	12	42.84
strofinaccio	MO	0	38	49172	51829.56	52265	51088.85	4	12	41.57
tagliaunghie	MO	NaN	16	10981	35408.22	12081	19490.07	4	12	40.72
tamburello	MO	0	73	10981	60408	34588	35325.67	4	10	36.91
tastiera	MO	2	259	10981	97769.80	65653	58134.60	3	8	27
telecomando	MO	1	551	25753	69158.88	36447	43786.29	5	11	41.91
temperino	MO	1	40	25753	87376.17	133168	82099.06	4	9	32.43
termometro	MO	0	154	25753	71501	48641	48631.67	4	10	39.45
trapano	MO	2	254	29265	84975.25	133168	82469.42	3	7	26.33
tubetto	MO	2	96	16862	52790.75	223642	97764.92	3	7	25.48
ventaglio	MO	0	139	25262	108094.67	52265	61873.89	3	9	31.41
volante	MO	1	1900	15502	122185	86456	74714.33	3	7	25.4
zuccheriera	MO	1	14	333	85319.88	65653	50435.29	4	11	39.45
angoscia	EMneg	1	401	42551	41736	57191	47159.33	3	8	28.87
antipatia	EMneg	1	61	42551	98048.50	57191	65930.17	5	9	28.79
avversione	EMneg	1	104	32484	92905.29	70433	65274.10	4	10	35.48
disagio	EMneg	0	1521	174105	45165.25	52265	90511.75	3	7	23.03
disgusto	EMneg	1	231	174105	44772.60	223642	147506.53	3	8	28.62
gelosia	EMneg	1	782	8939	61503.50	57191	42544.50	4	7	23.96
imbarazzo	EMneg	2	1989	14207	60728.67	7038	27324.56	4	9	31.92
infelicità	EMneg	0	212	107539	41897	93005	80813.67	5	10	29.8
inquietudine	EMneg	1	53	107539	36583.67	70433	71518.56	5	12	39.45
invidia	EMneg	3	517	107539	41897	57191	68875.67	3	7	20.15
malinconia	EMneg	1	181	56674	91237.57	57191	68367.52	5	10	34.12
nostalgia	EMneg	1	566	52903	77439.33	57191	62511.11	4	9	31.41
ostilità	EMneg	0	368	3806	82699.40	93005	59836.80	4	8	24.81
panico	EMneg	2	2866	45016	107347	26810	59724.33	3	6	21
rabbia	EMneg	3	3431	16575	23626.67	57191	32464.22	2	6	20.07
rammarico	EMneg	1	191	16575	77057.17	26810	40147.39	4	9	33.7

rancore	EMneg	1	895	16575	104412	133974	84987	3	7	27.09
repulsione	EMneg	0	65	23590	55560.14	70433	49861.05	4	10	35.22
rimorso	EMneg	3	679	51082	57015.50	40259	49452.17	3	7	26.08
rimpianto	EMneg	2	459	51082	92641.33	223642	122455.11	3	9	30.56
sdegno	EMneg	1	90	170	34449.33	133168	55929.11	2	6	23.45
sofferenza	EMneg	1	1445	57963	77515.57	25784	53754.19	4	10	36.66
terrore	EMneg	2	1148	25753	108271.80	133974	89332.93	3	7	26.59
tristezza	EMneg	1	1109	29265	67533.17	25784	40860.72	3	9	30.06
vergogna	EMneg	3	2628	25262	53131.80	61022	46471.93	3	8	31.41
affetto	EMpos	6	1602	5695	56892.50	223642	95409.83	3	7	24.89
allegria	EMpos	1	371	68073	65022.40	57191	63428.80	4	8	26.84
ammirazione	EMpos	1	272	11832	74967.13	70433	52410.71	5	11	38.69
divertimento	EMpos	1	2717	174105	99486.56	223642	165744.52	5	12	41.57
entusiasmo	EMpos	2	853	4561	62046.86	27069	31225.62	4	10	36
estasi	EMpos	2	311	26683	98875	28994	51517.33	3	6	19.47
euforia	EMpos	1	128	1839	63701.25	57191	40910.42	4	7	24.3
felicità	EMpos	1	2929	8694	87007.20	93005	62902.07	4	8	24.13
fiducia	EMpos	0	5456	25684	26870.25	57191	36581.75	3	7	21.84
gioia	EMpos	4	3090	28311	35936	57191	40479.33	2	5	15.66
gradimento	EMpos	1	257	14632	93770.29	223642	110681.43	4	10	37
gratitudine	EMpos	1	775	14632	86536.13	70433	57200.38	5	11	37.25
ilarità	EMpos	0	20	67973	98161.75	93005	86379.92	4	7	21
letizia	EMpos	0	62	38814	57952.75	57191	51319.25	3	7	20.48
meraviglia	EMpos	3	1939	28080	73575.57	57191	52948.86	4	10	33.6
ottimismo	EMpos	0	200	2888	61817.17	27069	30591.39	4	9	31.16
passione	EMpos	1	2456	45016	99446	70433	71631.67	3	8	28.11
riconoscenza	EMpos	1	163	51082	82940.33	25784	53268.78	5	12	44
serenità	EMpos	0	246	58313	123363	93005	91560.33	4	8	27.52
simpatia	EMpos	1	440	50691	79781	57191	62554.33	4	8	25.57
soddisfazione	EMpos	1	805	57963	54687.30	70433	61027.77	5	13	44.79
sollievo	EMpos	0	1189	57963	77574	12497	49344.67	3	8	27.09
stima	EMpos	5	742	49172	63435.50	22625	45077.50	2	5	16.93
tenerezza	EMpos	1	271	25753	88882	25784	46806.33	4	9	32.43
tranquillità	EMpos	0	454	29265	76285.11	93005	66185.04	4	12	39.54
abitudine	NEU	1	1455	9465	51751.50	70433	43883.17	5	9	29.38
buonsenso	NEU	0	187	4458	82890.33	40259	42535.78	3	9	34.71
cautela	NEU	1	426	46052	54334.50	78467	59617.83	3	7	24.98
contegno	NEU	2	135	135447	104467	133168	124360.67	3	8	31.41
criterio	NEU	0	117	9576	115488.80	52265	59109.93	3	8	26.33
decenza	NEU	3	269	116530	70689.25	25784	71001.08	3	7	25.99
discrezione	NEU	0	656	174105	73872.25	70433	106136.75	4	11	37.51
giudizio	NEU	0	2352	28311	110561	52265	63712.33	3	8	24.55
imparzialità	NEU	0	53	14207	71884.78	93005	59698.93	5	12	37.76
impressione	NEU	2	2782	14207	89438.50	70433	58026.17	4	11	38.02
memoria	NEU	2	5175	28080	74617.50	57191	53296.17	3	7	26
moderazione	NEU	0	106	29591	96005.88	70433	65343.29	5	11	40.47
neutralità	NEU	0	34	40713	76388.43	93005	70035.48	4	10	34.21
normalità	NEU	1	620	52903	78091.17	93005	74666.39	4	9	32.09

obiettività	NEU	0	66	1240	64806.50	93005	53017.17	5	11	33.95
opinione	NEU	1	3404	4461	94509	70433	56467.67	4	8	26.8
parsimonia	NEU	0	24	45016	78901.43	57191	60369.48	4	10	34.46
pensiero	NEU	1	4320	74312	102951	48641	75301.33	3	8	28.28
presentimento	NEU	1	785	66996	115006.20	223642	135214.73	5	13	47.07
prudenza	NEU	1	520	66996	53890.60	25784	48890.20	3	8	29.89
quotidianità	NEU	0	21	59294	68089.22	93005	73462.74	5	12	39.2
raziocinio	NEU	0	21	16575	62232.60	52265	43690.87	4	10	32
regolarità	NEU	0	89	23590	77927.71	93005	64840.90	5	10	35.22
senno	NEU	7	416	58313	113335	133168	101605.33	2	5	19
sobrietà	NEU	0	125	57963	47763.20	93005	66243.73	3	8	27.77

Supplementary Table 1B. Descriptive statistics of the rating scores for valence (val.), arousal (aro.), and imaginability (ima.) for every experimental stimulus obtained in the preliminary rating study on independent sample of 16 participants.

Word stimuli	Word Type	Mean val.	Sd val.	Mean aro.	Sd aro.	Mean ima.	Sd ima.
accendino	MO	0.06	0.44	3.25	1.24	7.00	0.00
anello	MO	0.56	0.63	2.81	1.38	6.88	0.34
annaffiatoio	MO	0.00	0.00	2.88	1.50	6.94	0.25
apriscatole	MO	0.13	0.34	3.00	1.41	6.81	0.75
spada	MO	0.0625	0.77	3.75	1.29	7.00	0.00
bastone	MO	-0.25	0.45	3.56	1.31	6.94	0.25
bicchiere	MO	0.38	0.62	3.00	1.41	7.00	0.00
bisturi	MO	-0.81	0.98	4.13	1.75	6.81	0.75
bollitore	MO	NaN	NaN	NaN	NaN	NaN	NaN
bottiglia	MO	0.19	0.54	3.00	1.37	7.00	0.00
bottone	MO	0.06	0.25	2.94	1.44	6.94	0.25
braccialetto	MO	NaN	NaN	NaN	NaN	NaN	NaN
bullone	MO	0.06	0.25	3.00	1.41	7.00	0.00
cacciavite	MO	0.13	0.34	3.06	1.44	6.88	0.50
caffettiera	MO	NaN	NaN	NaN	NaN	NaN	NaN
calcolatrice	MO	0.38	0.62	2.94	1.44	7.00	0.00
campanello	MO	0.06	0.25	3.19	1.47	6.94	0.25
cavatappi	MO	0.13	0.34	2.88	1.50	7.00	0.00
cellulare	MO	0.25	0.77	3.06	1.48	7.00	0.00
cerniera	MO	0.13	0.50	3.00	1.41	6.81	0.75
chitarra	MO	0.63	0.50	2.94	1.53	7.00	0.00
coltello	MO	-0.38	0.62	4.13	1.31	7.00	0.00
compasso	MO	0.19	0.54	2.88	1.50	7.00	0.00
computer	MO	0.50	1.03	3.13	1.54	7.00	0.00
coperchio	MO	0.06	0.25	2.88	1.50	6.94	0.25
cucchiaio	MO	0.06	0.25	3.06	1.44	7.00	0.00
fazzoletto	MO	0.13	0.34	2.94	1.34	6.81	0.75
fiammifero	MO	0.13	0.34	3.19	1.05	6.63	1.50
forbici	MO	0.00	0.37	3.25	1.18	6.75	1.00
forchetta	MO	0.19	0.40	2.88	1.50	6.94	0.25
fucile	MO	-1.31	1.14	4.19	1.56	6.94	0.25
grattugia	MO	0.06	0.57	2.88	1.50	7.00	0.00
grilletto	MO	-0.81	1.11	4.13	1.67	6.44	1.26
interruttore	MO	0.06	0.25	2.94	1.44	6.88	0.34
manette	MO	-1.00	0.82	4.25	1.39	6.94	0.25
manganello	MO	-1.50	1.10	4.38	1.31	6.88	0.50
maniglia	MO	0.06	0.25	3.06	1.44	6.81	0.75
mannaia	MO	-0.81	0.83	3.94	1.24	6.44	1.36
manopola	MO	0.00	0.00	3.00	1.41	6.69	0.79
martello	MO	0.13	0.50	3.13	1.41	6.94	0.25
matita	MO	0.69	0.70	2.69	1.54	7.00	0.00
mestolo	MO	0.19	0.54	2.88	1.50	7.00	0.00

orologio	MO	NaN	NaN	NaN	NaN	NaN	NaN
padella	MO	0.31	0.48	3.00	1.41	7.00	0.00
pennarello	MO	0.44	0.63	2.81	1.42	6.56	1.50
pennello	MO	0.31	0.48	2.88	1.50	6.69	1.25
pentola	MO	0.19	0.40	2.88	1.50	7.00	0.00
pettine	MO	-0.13	0.81	3.00	1.51	7.00	0.00
pinzatrice	MO	0.06	0.25	3.00	1.41	6.81	0.54
portafoglio	MO	0.06	0.44	3.13	1.41	6.81	0.75
pugnale	MO	-0.94	0.93	4.38	1.36	6.56	1.26
pulsante	MO	0.06	0.25	3.06	1.48	6.81	0.54
racchetta	MO	0.50	0.63	2.94	1.57	6.94	0.25
rasoio	MO	0.00	0.37	3.38	1.09	7.00	0.00
rastrello	MO	0.00	0.00	2.94	1.44	7.00	0.00
righello	MO	0.06	0.25	2.81	1.47	6.94	0.25
schiaccianoci	MO	0.00	0.37	2.81	1.47	6.94	0.25
scolapasta	MO	NaN	NaN	NaN	NaN	NaN	NaN
siringa	MO	-1.06	0.93	4.19	1.47	7.00	0.00
spatola	MO	0.19	0.54	2.88	1.50	6.63	0.89
spazzola	MO	0.06	0.68	3.00	1.41	6.81	0.75
spazzolino	MO	0.13	0.50	2.94	1.44	7.00	0.00
spremiagrumi	MO	0.38	0.62	2.75	1.39	6.81	0.54
strofinaccio	MO	0.00	0.52	2.88	1.50	6.88	0.50
tagliaunghie	MO	0.13	0.34	3.00	1.41	6.81	0.75
tamburello	MO	0.38	0.62	3.00	1.37	7.00	0.00
tastiera	MO	0.25	0.58	3.13	1.54	7.00	0.00
telecomando	MO	0.19	0.54	3.13	1.36	7.00	0.00
temperino	MO	0.19	0.40	2.88	1.50	7.00	0.00
termometro	MO	-0.19	0.83	3.25	1.48	7.00	0.00
trapano	MO	-0.13	0.62	3.63	1.86	7.00	0.00
tubetto	MO	0.00	0.00	2.88	1.50	6.75	1.00
ventaglio	MO	0.31	0.48	2.94	1.34	6.81	0.54
volante	MO	0.31	0.79	2.75	1.34	7.00	0.00
zuccheriera	MO	NaN	NaN	NaN	NaN	NaN	NaN
angoscia	EMneg	-2.81	0.40	5.81	1.52	3.13	1.89
antipatia	EMneg	-1.75	0.45	4.81	0.98	3.06	1.53
avversione	EMneg	-1.81	1.11	4.75	0.93	3.06	1.61
disagio	EMneg	-2.38	0.72	4.75	1.53	2.75	1.81
disgusto	EMneg	-2.25	0.58	4.56	0.81	3.75	1.39
gelosia	EMneg	-1.75	1.00	5.19	1.17	3.81	1.97
imbarazzo	EMneg	-1.38	1.26	4.94	1.29	3.50	1.83
infelicità	EMneg	-2.44	0.63	4.56	1.82	3.75	1.69
inquietudine	EMneg	-2.31	0.79	5.38	1.50	2.56	1.75
invidia	EMneg	-2.19	0.98	5.25	1.06	3.31	1.82
malinconia	EMneg	-1.81	0.91	3.44	1.67	2.81	1.47
nostalgia	EMneg	-1.81	0.98	3.81	1.33	2.63	1.82
ostilità	EMneg	-2.06	0.85	5.69	1.25	3.00	1.75
panico	EMneg	-2.63	0.62	6.63	0.81	4.13	1.78
rabbia	EMneg	-1.88	1.31	5.75	1.18	4.31	1.66

rammarico	EMneg	-1.69	0.70	4.44	0.89	2.38	1.54
rancore	EMneg	-2.25	1.13	5.44	1.15	2.75	1.73
repulsione	EMneg	-1.88	0.72	4.75	1.00	3.38	1.67
rimorso	EMneg	-2.19	0.83	4.75	1.06	2.19	1.52
rimpianto	EMneg	-2.19	0.83	4.44	1.21	2.50	1.75
sdegno	EMneg	-1.69	1.14	4.75	0.45	3.00	1.67
sofferenza	EMneg	-2.50	0.82	5.13	1.41	4.06	1.65
terrore	EMneg	-2.63	0.62	6.25	1.18	4.06	1.34
tristezza	EMneg	-2.19	0.75	3.81	1.64	3.88	1.26
vergogna	EMneg	-2.19	0.83	5.25	1.06	3.56	1.75
affetto	EMpos	2.38	0.81	2.25	1.44	3.44	2.06
allegria	EMpos	2.38	0.72	3.94	1.98	4.00	1.46
ammirazione	EMpos	1.75	0.86	2.94	1.34	2.56	1.59
divertimento	EMpos	2.31	0.79	4.31	1.49	3.88	1.96
entusiasmo	EMpos	2.19	0.75	4.75	1.81	3.75	1.84
estasi	EMpos	1.75	1.29	3.00	1.71	3.63	1.86
euforia	EMpos	1.75	1.18	5.38	1.54	3.88	1.67
felicità	EMpos	2.56	0.51	3.75	1.88	4.06	1.88
fiducia	EMpos	2.25	0.77	1.88	1.26	2.44	1.63
gioia	EMpos	2.50	0.73	4.06	1.88	4.19	1.80
gradimento	EMpos	1.44	0.96	2.50	1.26	2.81	1.76
gratitudine	EMpos	1.94	0.77	2.44	1.36	2.56	1.75
ilarità	EMpos	1.38	1.41	4.25	1.53	3.25	2.08
letizia	EMpos	1.38	1.09	2.38	1.36	2.88	1.71
meraviglia	EMpos	2.63	0.62	4.00	1.55	3.50	1.51
ottimismo	EMpos	1.81	1.22	2.44	1.21	2.69	1.96
passione	EMpos	2.19	0.83	5.44	1.63	4.13	1.71
riconoscenza	EMpos	1.88	0.81	2.25	1.06	2.63	2.00
serenità	EMpos	2.25	0.68	1.44	0.89	3.31	1.89
simpatia	EMpos	2.00	0.82	3.19	1.17	2.81	1.68
soddisfazione	EMpos	2.13	0.81	2.06	1.12	3.38	2.25
sollievo	EMpos	2.00	0.73	1.50	0.73	3.25	2.11
stima	EMpos	1.88	0.89	2.56	1.46	2.44	1.90
tenerezza	EMpos	2.13	0.62	1.50	0.63	3.81	1.64
tranquillità	EMpos	1.75	0.86	1.25	0.77	3.06	1.44
abitudine	NEU	0.00	1.10	2.13	1.45	2.38	1.96
buonsenso	NEU	1.38	0.96	2.38	1.15	1.69	1.30
cautela	NEU	0.25	0.77	3.13	1.36	2.13	1.26
contegno	NEU	0.25	0.86	2.81	1.22	2.00	1.15
criterio	NEU	0.69	0.95	2.88	1.31	1.44	0.73
decenza	NEU	0.13	1.15	2.69	1.20	2.06	1.44
discrezione	NEU	0.88	0.81	2.44	1.21	1.75	1.29
giudizio	NEU	-0.25	1.24	3.94	1.77	2.44	1.71
imparzialità	NEU	0.81	0.98	2.81	1.47	2.00	1.41
impressione	NEU	0.25	0.68	3.88	1.41	1.69	1.40
memoria	NEU	1.06	1.29	2.88	1.31	2.25	1.61
moderazione	NEU	0.38	0.96	2.38	1.31	1.81	1.17
neutralità	NEU	-0.13	0.96	3.00	1.32	1.81	1.05

normalità	NEU	0.44	0.73	2.31	1.30	2.00	1.32
obiettività	NEU	0.88	0.89	2.31	1.25	1.81	1.47
opinione	NEU	0.94	1.12	3.75	1.53	2.31	2.09
parsimonia	NEU	0.13	1.09	2.81	1.28	1.81	1.11
pensiero	NEU	1.13	1.41	3.13	1.89	2.38	1.67
presentimento	NEU	-0.94	0.77	4.75	1.24	2.13	1.67
prudenza	NEU	0.56	0.63	3.06	1.34	2.13	1.31
quotidianità	NEU	0.56	0.81	2.31	1.20	2.75	2.08
raziocinio	NEU	1.25	1.06	2.56	1.21	1.88	1.31
regolarità	NEU	0.38	0.89	1.75	1.06	2.44	1.55
senno	NEU	1.00	1.03	2.44	1.21	1.69	1.08
sobrietà	NEU	0.31	0.95	2.50	1.21	2.69	1.82

Supplementary Table 2A. 100 words referring to jobs and careers used in the training session.

1.	allenatore	51.	ginecologa
2.	archeologa	52.	giornalista
3.	architetto	53.	giudice
4.	astronauta	54.	hostess
5.	astronomo	55.	idraulico
6.	attore	56.	imbianchino
7.	bagnina	57.	infermiera
8.	balia	58.	ingegnere
9.	ballerina	59.	insegnante
10.	banchiere	60.	macchinista
11.	barbiere	61.	macellaio
12.	barista	62.	magistrato
13.	benzinaio	63.	maresciallo
14.	biologa	64.	minatore
15.	calciatore	65.	modella
16.	calzolaio	66.	neurologa
17.	cameriera	67.	notaio
18.	camionista	68.	oste
19.	cantante	69.	ostetrica
20.	carabiniere	70.	parrucchiera
21.	cardiologa	71.	pasticcere
22.	carpentiere	72.	pediatra
23.	casalinga	73.	pescatore
24.	cassiere	74.	pilota
25.	centralinista	75.	pittore
26.	chef	76.	poeta
27.	chitarrista	77.	pompiere
28.	ciclista	78.	postino
29.	commessa	79.	prestigiatore
30.	contabile	80.	prete
31.	contadino	81.	psicologa
32.	coreografa	82.	ragioniere
33.	dentista	83.	redattore
34.	deputato	84.	regista

35. disinfestatore
36. economista
37. elettricista
38. estetista
39. fabbro
40. falegname
41. farmacista
42. filosofa
43. fioraia
44. fisioterapista
45. fornaio
46. fotografa
47. fruttivendolo
48. gelataio
49. geologa
50. giardiniere

85. rettore
86. ricercatore
87. sarta
88. scenografo
89. sceriffo
90. scrittrice
91. scultore
92. segretaria
93. senatore
94. sindaco
95. soldato
96. stilista
97. tassista
98. tipografo
99. truccatrice
100. usciere

Supplementary Table 2B. Lexical and sublexical variables of training stimuli referring to jobs and careers, matched with manipulable objects (MO) and emotion (EM, with negatively valenced [EMneg], positively valenced [EMpos], and neutral [NEU]) word stimuli, mean (sd).

Variable	MO	EMneg	EMpos	NEU	Career	Kruskal-Wallis	*p* value
Lexical frequency	975.4 (1663)	879.7 (949.1)	1109.9 (1319.3)	966.3 (1462.2)	770.43 (1254.557)	5.6171	0.2296
N. syllables	3.48 (0.67)	3.56 (0.87)	3.8 (0.87)	3.8 (0.91)	3.69 (0.87)	6.8592	0.143
N. letters	8.76 (1.8)	8.32 (1.55)	8.72 (2.23)	9.2 (1.91)	8.8 (2.01)	2.7892	0.56

Supplementary Table 3A. 100 words referring to living/natural entities used to assess participants' perceptual threshold.

1. acquazzone
2. agnello
3. agrifoglio
4. albero
5. alligatore
6. anatra
7. anguilla
8. aragosta
9. armadillo
10. avvoltoio
11. balena
12. barboncino
13. camaleonte
14. cammello
15. campagna
16. canarino
17. canguro
18. castoro
19. cavalletta
20. cavallo
21. cespuglio
22. ciliegio
23. cinghiale
24. civetta
25. coccinella
26. coccodrillo
27. colomba
28. coniglio
29. cornacchia
30. costellazione
31. criceto
32. crisantemo
33. delfino
34. deserto
35. dinosauro
36. elefante
37. farfalla
38. foresta
39. formica
40. furetto
41. gabbiano
42. galassia
43. gallina
44. gamberetto
45. gelsomino
46. girasole
47. gorilla
51. ippopotamo
52. lavanda
53. libellula
54. lucertola
55. magnolia
56. maiale
57. margherita
58. marmotta
59. montagna
60. nebulosa
61. oceano
62. orchidea
63. ornitorinco
64. pantera
65. pappagallo
66. pecora
67. pellicano
68. pettirosso
69. pianeta
70. pinguino
71. pioggia
72. pipistrello
73. procione
74. pterodattilo
75. pulcino
76. quadrifoglio
77. quercia
78. rinoceronte
79. rosmarino
80. salamandra
81. sanguisuga
82. scarabeo
83. scarafaggio
84. sciacallo
85. scimmia
86. scoiattolo
87. serpente
88. spiaggia
89. squalo
90. tacchino
91. tartaruga
92. tempesta
93. temporale
94. testuggine
95. tirannosauro
96. tricheco
97. uccello

48. granchio
49. insalata
50. insetto
98. universo
99. usignolo
100. vitello

Supplementary Table 3B. Lexical and sublexical and psycholinguistic variables of the stimuli referring living/natural items used for assessing the participants' perceptual threshold, matched with manipulable objects (MO) and emotion (EM, with negatively valenced [EMneg], positively valenced [EMpos], and neutral [NEU]) word stimuli, mean (sd).

Variable	MO	EMneg	EMpos	NEU	Living	Kruskal-Wallis	*p* value
Lexical frequency	975.4 (1663)	879.7 (949.1)	1109.9 (1319.3)	966.3 (1462.2)	844.74 (1372)	5.2586	0.262
First bigram frequency	36399.6 (32386.9)	51354 (47659.77)	37908.56 (35402.45)	47136 (42814.64)	40578.68 (38356.7)	2.3387	0.6737
Last bigram frequency	70599.56 (49678.53)	76472.72 (55314.3)	75793.48 (60578.25)	79331.6 (40676.11)	72701.65 (48623.17)	2.3375	0.674
Medial bigrams frequency	74687.16 (22995.85)	67007 (25336.21)	75560.52 (21534.27)	81195.73 (20707.4)	71242.46 (23138.97)	6.4627	0.1672
Mean bigram frequency	60562.12 (20598.21)	64944.58 (27122.55)	63087.52 (29200.52)	69221.12 (23766.04)	61507.6 (23470.87)	2.6986	0.601
N. orthographic neighbours	1.51 (1.51)	1.32 (0.94)	1.36 (1.58)	0.92 (1.52)	1.4 (1.188)	7.7002	0.103
N. syllables	3.48 (0.67)	3.56 (0.87)	3.8 (0.87)	3.8 (0.91)	3.5 (0.76)	6.2544	0.181
N. letters	8.76 (1.8)	8.32 (1.55)	8.72 (2.23)	9.2 (1.91)	8.43 (1.55)	4.7944	0.309

Supplementary Figure 1.

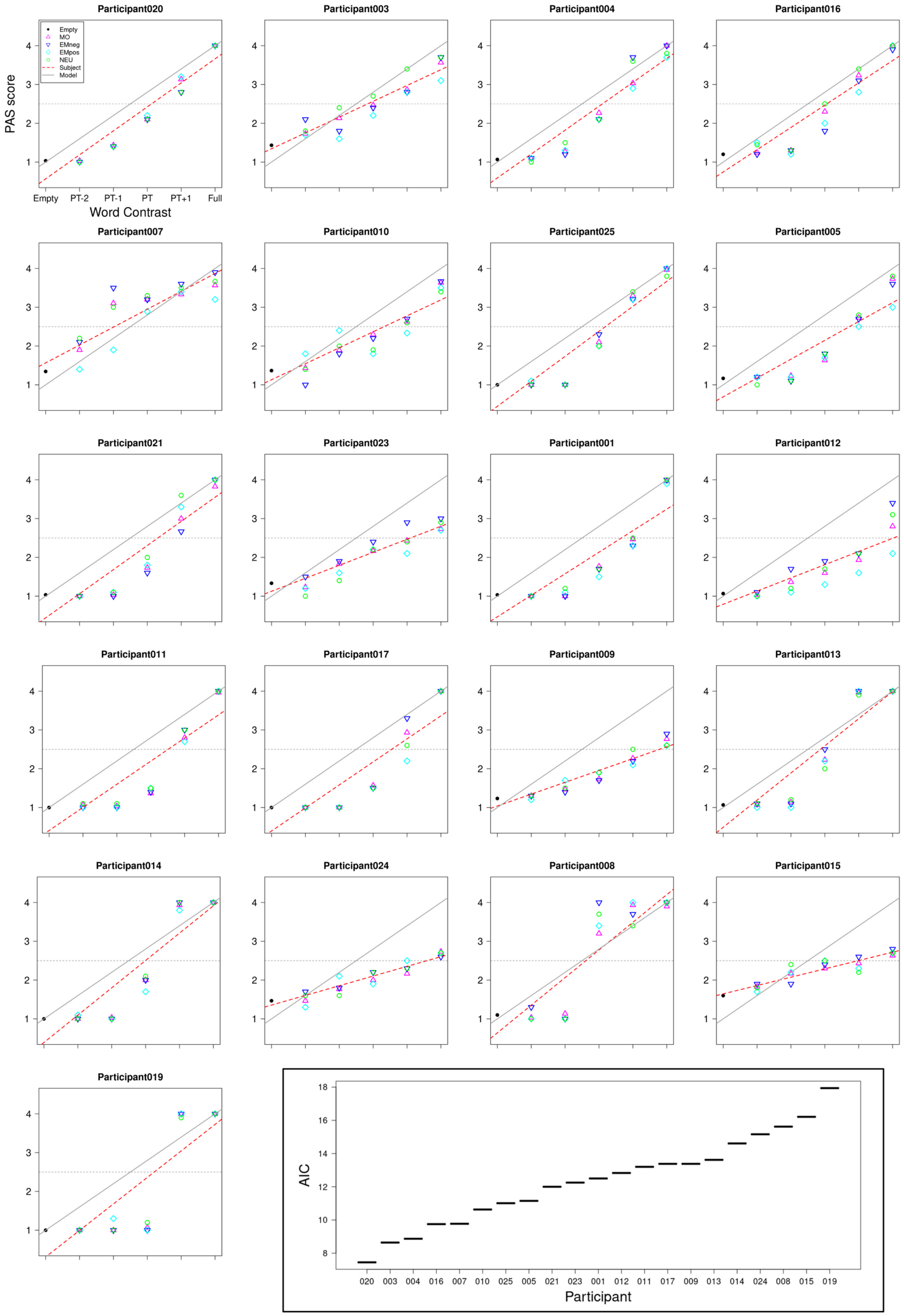

Legend to Supplementary Figure 1. **Selection of participants for the BOLD analysis as a function of PAS**. The participants were ranked according to the Akaike information criterion (AIC; bottom right inset), obtained by Maximum Likelihood Estimation of the fit of the individual Word Contrast-to-PAS linear function (red dashed line; calculated on the average PAS ratings across all stimulus types (Empty, MO, EMneg, EMpos, NEU)) to a Word Contrast-to-PAS linear model function (gray line). The conventions of the dot-plots of all participants are as displayed for the "Participant020" on top left. None of the participants markedly departed from a linear increase of PAS score with increasing contrast level. However, "Participant012" was excluded from the analyses of BOLD activation as a function of PAS rating, because of an exceedingly low number of PAS{3,4} responses. This left the sub-sample for these analyses with 20 participants.
